# Thyrotoxic Hypokalemic Periodic Paralysis in Colombia: From a Single Case to a Review of the National Experience

**DOI:** 10.3390/diseases14090345

**Published:** 2026-09-17

**Authors:** H. A. Nati-Castillo, Alin Abreu Lomba, Juan David Urrea Ospina, Jhan S. Saavedra-Torres, Sebastián Lozano Ciro, Juliana Vela Paz, Nelly Johana Alvis Linero, Juan S. Izquierdo-Condoy

**Affiliations:** 1 School of Health Sciences, Universidad CES, Medellin 050021, Colombia; 2 School of Health Sciences, Universidad Libre, Cali 760031, Colombia; 3 Endocrinology Department, Clínica Imbanaco, Cali 760042, Colombia; 4 School of Health Sciences, Institución Universitaria Visión de las Américas, Pereira 660003, Colombia; 5 Facultad de Ciencias de la Salud, Universidad ICESI, Cali 760031, Colombia; 6 Facultad de Ciencias de la Salud, Universidad del Sinu, Cartagena 130001, Colombia; 7 One Health Research Group, Universidad de las Américas, Quito 170124, Ecuador

**Keywords:** hypokalemia, thyrotoxicosis, periodic paralysis, Graves disease, endocrine emergency

## Abstract

Thyrotoxic periodic paralysis (TPP) is a reversible but potentially severe complication of thyrotoxicosis characterized by acute muscle weakness and hypokalemia resulting predominantly from intracellular potassium redistribution. Although classically associated with Asian populations, TPP is increasingly recognized in Latin America. We report a 38-year-old Colombian man with recurrent nocturnal proximal quadriparesis, preserved deep tendon reflexes, serum potassium of 2.3 mEq/L, and biochemical thyrotoxicosis secondary to Graves disease. In parallel, we conducted a structured review of published Colombian cases identified through searches of PubMed/MEDLINE, Scopus, Web of Science, SciELO, and Google Scholar from inception through 1 May 2026. Fourteen previously published cases were identified, yielding 15 cases including the index patient. All patients were male, with a mean age of 31.4 ± 7.4 years. Deep tendon reflexes were normal in 4/13 (30.8%) cases in which they were documented, demonstrating that preserved reflexes do not exclude TPP. TSH was suppressed below 0.05 mIU/L in 14/15 (93.3%) patients. Electrocardiographic findings were reported in only 7/15 cases, and antithyroid drug doses in 6/15, highlighting substantial heterogeneity in reporting. All reported patients recovered muscle strength following acute management. TPP should therefore be considered in patients presenting with acute proximal weakness and hypokalemia even when deep tendon reflexes are preserved or overt manifestations of thyrotoxicosis are absent. Standardized reporting of biochemical, electrocardiographic, therapeutic, and follow-up data is needed to improve characterization of TPP in underrepresented populations.

## 1. Introduction

Thyrotoxic periodic paralysis (TPP) is a reversible but potentially severe neuromuscular complication of thyrotoxicosis, characterized by acute episodes of flaccid muscle weakness associated with hypokalemia resulting predominantly from an intracellular shift of potassium [1,2,3]. Although uncommon in Western populations, TPP has been classically described in Asian populations, and predominantly affects young men despite the higher overall prevalence of thyroid disease among women [1,4].

Clinically, weakness typically involves the proximal muscles and lower limbs, although progression to quadriparesis and, less frequently, respiratory muscle involvement may occur [1,4]. Because sensory function and consciousness are generally preserved, the presentation can mimic other metabolic, neurologic, or hereditary causes of acute paralysis. Differentiation from familial hypokalemic periodic paralysis relies on the demonstration of thyrotoxicosis and the exclusion of alternative causes of potassium depletion [2,5,6]. Acute management focuses on cautious correction of hypokalemia and control of the hyperadrenergic state, whereas definitive control of the underlying thyrotoxicosis is essential to prevent recurrence [2,5,6].

Although TPP has been increasingly recognized outside traditionally affected populations, its clinical characteristics and reporting patterns in Latin America remain incompletely characterized [7,8]. Published Colombian experience consists predominantly of isolated case reports and small case series, limiting an integrated assessment of its clinical, biochemical, electrocardiographic, and therapeutic features. Therefore, we report a Colombian patient with recurrent TPP associated with Graves disease and conduct a structured review of published Colombian cases to characterize their clinical presentation, biochemical and electrocardiographic findings, management, outcomes, and completeness of reporting.

## 2. Literature Search and Data Extraction

A structured literature search was conducted to identify previously published cases of thyrotoxic periodic paralysis in Colombia. PubMed/MEDLINE, Scopus, Web of Science, SciELO, and Google Scholar were searched from database inception through 1 May 2026. Search terms included combinations of “thyrotoxic periodic paralysis”, “thyrotoxic hypokalemic periodic paralysis”, “hypokalemic periodic paralysis”, “Colombia”, and “Colombian”, together with their Spanish-language equivalents, including “parálisis periódica tirotóxica”, “parálisis periódica hipocalémica tirotóxica”, and “parálisis periódica hipopotasémica tirotóxica”. Reference lists of eligible publications were also examined to identify additional reports.

Reports were eligible if they described one or more patients diagnosed with thyrotoxic periodic paralysis in Colombia and provided sufficient individual-level clinical or biochemical information for case-level extraction. Reports describing patients outside Colombia, duplicate publications, reviews without extractable individual patient data, and reports in which the diagnosis of thyrotoxic periodic paralysis could not be established were excluded.

For each eligible case, available data were extracted on age, geographic location, pattern of muscle weakness, deep tendon reflexes, serum potassium, magnesium, thyroid function, electrocardiographic findings, treatment, and requirement for intensive care. Data were recorded as reported in the original publications without imputation of missing values. When a variable was not reported, it was classified as not specified or not reported, as appropriate. One report for which only the published abstract could be accessed was retained, and data derived from that source were explicitly identified as not independently verifiable from the full text.

The findings were synthesized descriptively. Continuous variables were summarized using means and standard deviations when appropriate, whereas categorical variables were reported as counts and proportions using available-case denominators. Because of the small number of published cases and heterogeneity in reporting, no inferential statistical analyses were performed.

## 3. Case Presentation

38-year-old man described in the medical record as Mestizo, a broad sociocultural category commonly used in Colombia for individuals of mixed ancestry, from Palmira, a city in southwestern Colombia, with no relevant medical history, presented to the emergency department with an 18-h history of progressive, symmetrical proximal weakness that began in the lower limbs during sleep and subsequently extended to the upper limbs. He denied sensory disturbances, sphincter dysfunction, or altered consciousness. On review of systems, he reported two previous self-limited episodes with similar characteristics during the preceding year, for which he had not sought medical attention. He had no family history of neuromuscular disease and denied medication or substance use. He reported unintentional weight loss over the preceding months, with a lowest documented weight of 95 kg for a height of 186 cm, together with diaphoresis and insomnia. His baseline weight and the exact magnitude of weight loss were not available. Heat intolerance and other hyperthyroid symptoms were not documented in the available medical record. He had stopped smoking seven years earlier and did not consume alcohol. No carbohydrate-rich meal, strenuous exercise, acute infection, medication exposure, or other recognized precipitant preceded the episode; recent emotional stress was not documented.

On physical examination, he was alert and oriented, with a blood pressure of 139/78 mmHg, a heart rate of 112 beats per minute, a temperature of 36.7 °C and no signs of heart failure. Neither goiter, exophthalmos, nor distal tremor was present. Neurologic examination revealed symmetrical, predominantly proximal quadriparesis, with muscle strength of 4/5 in the upper limbs and 3/5 in the lower limbs according to the Medical Research Council (MRC) scale. Muscle tone was normal, deep tendon reflexes were symmetrical and graded 2+, and there were no sensory deficits or cranial nerve, cerebellar, or pyramidal signs. Initial laboratory testing showed a serum potassium level of 2.3 mEq/L, magnesium of 1.6 mEq/L, phosphate of 2.9 mg/dL, and normal renal function (Table 1). Electrocardiography showed a markedly prolonged corrected QT (QTc) interval (649 ms), flattened T waves, and prominent U waves, findings consistent with severe hypokalemia. Arterial blood gas analysis showed no acid-base or oxygenation abnormalities. Twenty-four-hour urinary potassium excretion was low (14 mEq/day) (Table 1), compatible with renal potassium conservation. In the absence of gastrointestinal losses or acid-base disturbances, these findings favored intracellular potassium redistribution.

The combination of recurrent symmetrical proximal quadriparesis, preserved muscle tone and deep tendon reflexes, and the absence of sensory or pyramidal involvement suggested a metabolic or electrolyte-related myopathic syndrome, with hypokalemic periodic paralysis as the leading diagnostic consideration. Although the sensory examination was normal, nerve conduction studies showed electrophysiological signs of a demyelinating sensorimotor polyneuropathy involving the lower limbs, without evidence of lumbosacral root involvement.

Given the suspicion of an endocrine cause, thyroid function testing was performed and showed a TSH level < 0.005 mIU/L and free T4 > 7.7 ng/dL (Table 1), consistent with thyrotoxicosis. Thyroid scintigraphy demonstrated diffusely increased uptake, and anti-TSH receptor antibodies were positive, confirming Graves disease as the underlying etiology.

The diagnosis of thyrotoxic periodic paralysis was established based on the characteristic triad of acute muscle weakness, hypokalemia, and biochemical thyrotoxicosis. The clinical presentation was also consistent with commonly described diagnostic features, including male sex, nocturnal onset of symmetrical flaccid weakness lasting more than two hours, serum potassium < 3.5 mEq/L, absence of an alternative apparent cause, and confirmed thyrotoxicosis.

Because of the severity of the electrolyte disturbance, the patient was initially admitted to the critical care unit, where he received intravenous potassium replacement, first through a peripheral line and subsequently through a central venous catheter. The exact cumulative intravenous dose and infusion rates could not be reconstructed from the available medical record. Serum potassium increased from 2.3 mEq/L at admission to 4.6 mEq/L on the following day, accompanied by progressive recovery of muscle strength. No rebound hyperkalemia was documented in the available measurements. Propranolol was initiated before muscle strength had fully recovered, although the exact time of administration relative to potassium normalization was not available. Oral potassium chloride 10% solution (100 mg/mL) was subsequently prescribed at 5 mL every 8 h for seven days, equivalent to 500 mg, or approximately 6.7 mEq, per dose.

Treatment of thyrotoxicosis was initiated with methimazole 10 mg every 12 h and propranolol 40 mg every 8 h. Separately, neurology administered intravenous methylprednisolone 1 g daily for five days because nerve conduction studies had shown findings interpreted as a demyelinating sensorimotor polyneuropathy involving the lower limbs. This was followed by oral prednisone 50 mg/day with gradual dose tapering over the subsequent months. The exact tapering schedule and total duration of glucocorticoid therapy were not available. Glucocorticoids were not administered for TPP, thyrotoxicosis, or suspected thyroid storm.

The patient was discharged after an eight-day hospitalization. Oral potassium chloride 10% solution (100 mg/mL) was prescribed at 5 mL every 8 h for seven days. At the only available post-discharge endocrinology assessment, approximately three months later, TSH remained suppressed (<0.005 mIU/L). Follow-up free T4 and serum potassium measurements were unavailable. No further episodes of muscle weakness had been documented by that visit. Information on the exact antithyroid and beta-blocker doses being taken at the three-month assessment was not available. Thyroidectomy had been proposed as definitive therapy; however, authorization was denied by the patient’s health insurer.

## 4. Discussion

**Figure 1:**
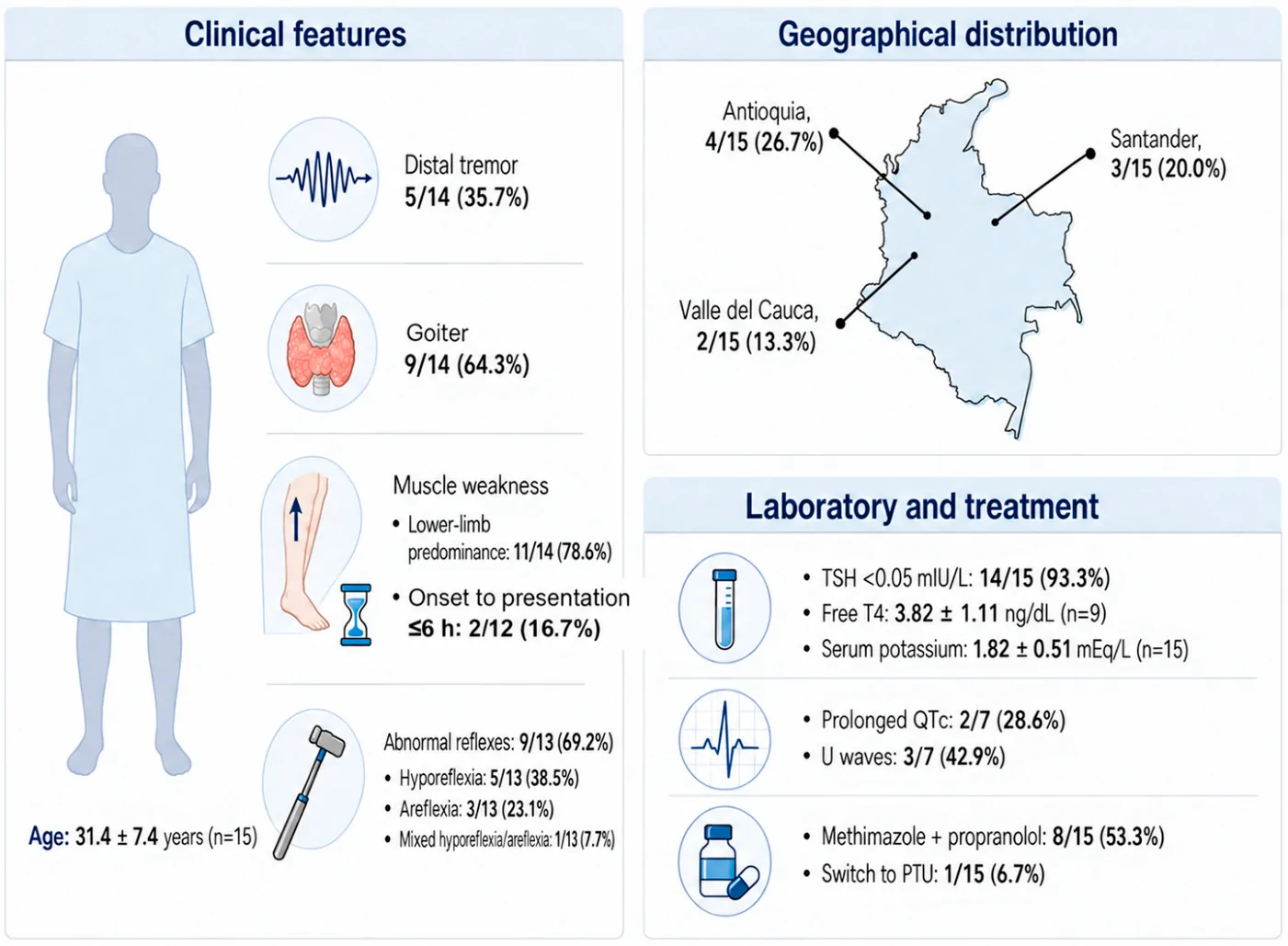
Clinical, geographical and therapeutic profile of the 15 Colombian cases of thyrotoxic periodic paralysis. The left panel summarizes clinical characteristics, including age, distal tremor, goiter, pattern of weakness, time from onset of weakness to presentation, and deep tendon reflexes. Among the 13 cases with documented deep tendon reflexes, five had hyporeflexia, three had areflexia, one had a mixed pattern of hyporeflexia and areflexia, and four had preserved reflexes. The upper-right panel highlights the three departments that together accounted for 60% of the cases. The lower-right panel summarizes laboratory, electrocardiographic, and treatment findings. Denominators vary across variables because not every primary report documented each item. Time from onset of weakness to presentation was defined as the interval between the onset of muscle weakness and presentation to the emergency department. This interval was reported in 12 of the 15 cases, of which two presented within six hours. Source: prepared by the authors.

Although TPP was classically described in Asian populations, reports among Hispanic patients have become increasingly frequent [7]. In the present review of 15 Colombian cases, including the index case (Table 2), all patients were male, with a mean age of 31.4 ± 7.4 years (range, 20–44 years), comparable to the mean age of 27 years reported among Hispanic patients in a previous series [8]. Antioquia accounted for 4/15 cases (26.7%), Santander for 3/15 (20.0%), and Valle del Cauca for 2/15 (13.3%), together representing 60% of the published cases (Figure 1). In the absence of a national registry, however, this geographical distribution should be interpreted as a description of the published literature rather than as an estimate of the true regional burden of TPP in Colombia.

The neuromuscular phenotype was broadly consistent with that described in the literature: 11/14 (78.6%) verifiable cases showed predominantly lower-limb weakness, with ascending progression to the upper limbs in six cases [1,9]. Deep tendon reflexes were abnormal in 9/13 (69.2%) cases in which they were documented, whereas 4/13 (30.8%) had preserved reflexes. This finding is clinically relevant because normal deep tendon reflexes do not exclude TPP [1,9]. Goiter was documented on physical examination or imaging in 9/14 (64.3%) verifiable cases, and distal tremor in 5/14 (35.7%). Although these findings may raise suspicion of an underlying thyroid disorder, their absence should not preclude thyroid function testing in patients presenting with acute hypokalemic weakness.

Mean serum potassium at presentation was 1.82 ± 0.51 mEq/L (range, 0.68–3.10 mEq/L), lower than the mean value of 2.3 ± 0.75 mEq/L reported in a Japanese reference series [10]. This difference should be interpreted cautiously given the small number of Colombian cases and the publication-based nature of the present dataset. Serum magnesium was reported in only 4/15 cases and was within the reported reference range in all four, with a mean value of 1.76 mEq/L. The limited number of measurements precludes meaningful assessment of the role of magnesium abnormalities in this population.

The thyroid profile was characterized by marked TSH suppression, with values below 0.05 mIU/L in 14/15 patients (93.3%). Among the nine independently verifiable cases with exact quantitative free T4 measurements, mean free T4 was 3.82 ± 1.11 ng/dL. The three cases reported by Ladino-Malagón et al. provided total rather than free thyroxine values and were therefore not included in this calculation. The index case was also excluded from the mean because free T4 was reported only as greater than the assay upper limit (>7.7 ng/dL), rather than as an exact quantitative value. These findings illustrate the biochemical heterogeneity of published case reporting despite the consistent presence of thyrotoxicosis.

Electrocardiographic findings were described in only 7/15 cases, precluding reliable estimation of the frequency of cardiac electrical abnormalities. Among those with reported ECG findings, 2/7 (28.6%) had QTc prolongation, 3/7 (42.9%) had U waves, one had second-degree Mobitz I atrioventricular block, and one had a normal ECG; no cases of atrial fibrillation were reported. Given the potential for severe hypokalemia to produce clinically significant electrical instability, the limited and inconsistent reporting of ECG findings represents an important gap in the Colombian literature and supports systematic electrocardiographic assessment in future reports.

Treatment patterns were generally consistent with conventional management of TPP, although reporting detail varied substantially across publications. Methimazole and propranolol were explicitly reported in 8/15 cases (53.3%), whereas two additional reports described the use of antithyroid drugs and beta-blockers without specifying the agents. Methimazole doses were provided in only six cases, reported in five publications, with a mean reported dose of 24.2 ± 9.2 mg/day. One patient was switched to propylthiouracil because of a cutaneous adverse reaction to methimazole. Five patients (33.3%) required intensive care, primarily because of severe hypokalemia or respiratory compromise. Graves disease was documented as the underlying etiology in eight cases, whereas three reports described thyroiditis on ultrasonography, and the remaining reports did not clearly establish the cause of thyrotoxicosis. This variability contrasts with Asian series in which Graves disease is the predominant underlying disorder [11]. In the series by Karndumri et al., paralysis was the first manifestation of Graves disease in 82.5% of 63 patients, with a median serum potassium level of 2.1 mmol/L [12].

**Table 2:** Clinical, laboratory and management features of the 15 reported Colombian cases of thyrotoxic periodic paralysis (including the index case).

Case (Author, Year)	Age (Years)	Department	Pattern of Weakness	Time from Onset of Weakness to Presentation (as Reported)	Reflexes on Admission	K^+^ (mEq/L)	Mg^2+^ (mEq/L)	Free T4 (ng/dL)	TSH (mIU/L)	ECG Findings	Treatment (Dose)	ICU
Duarte et al., 2011 [13]	39	Santander	Flaccid quadriparesis (LL 1/5, UL 2/5); second episode	6 h (second episode; onset at 01:00 during sleep)	Patellar areflexia	1.46 (4.08 in the first episode)	NR	3.25 (41.8 pmol/L)	<0.05	Flattened T wave	Corticosteroid + beta-blocker (no dose); I-131 30 mCi	No
Ladino-Malagón et al., 2012 (C1) [14]	27	Antioquia	Recurrent quadriplegia on waking	NR (recurrent episodes on waking, resolving in 12–48 h)	Symmetrical hyporeflexia	1.5	NR	NR (total T4 4.7)	Undetectable	NR	IV potassium + beta-blocker + antithyroid drug (neither named nor dosed)	No
	20	Antioquia	Generalized quadriparesis 2/5	NR (onset on waking)	UL hyporeflexia and LL areflexia	2.1	1.8	NR (total T4 3.26)	Undetectable	NR	IV potassium (drugs not specified)	No
	24	Antioquia	Progressive quadriparesis, LL 2/5 > UL 3/5	24 h	Generalized areflexia	1.7	NR	NR (total T4 6.4)	Undetectable	NR	IV potassium (drugs not specified)	No
Acevedo Rueda and Rincón Albarrán, 2013 [15]	20	Santander	Proximal paraparesis (LL 1/5) + paresthesia in all four limbs	2 h	Hyporeflexia	1.9	1.56	4.6	0.006	Sinus tachycardia	Potassium replacement; I-131 + propranolol (no dose)	No
Ochoa Morón and Pérez Rodríguez, 2013 [16]	31	Atlántico	Asymmetric quadriparesis, LL (1-2/5) > UL (2-3/5)	12 h	Areflexia (0/++++)	1.74	NR	3.60	<0.005	Mobitz I AV block + supraventricular ectopics	Methimazole 30 mg/day + propranolol 40 mg every 12 h	No
Pinzón and Vásquez, 2014 [17]	29	Huila	Ascending quadriplegia (1/5 in all four limbs)	11 h	Generalized hyporeflexia	0.68	NR	2.38 (30.7 pmol/L)	<0.05	Flattened T wave, U wave, QTc 500 ms	IV potassium; antithyroid drug + beta-blocker + potassium gluconate (no dose); I-131	Yes
Cogollo González et al., 2016 [18]	38 *	Bolívar	Quadriparesis *	NV *	NV *	1.73 *	1.7 *	4.22 *	0.002 *	Sinus tachycardia *	Methimazole + propranolol (no dose); IV potassium through a central line	Yes
Moreno-Rozo et al., 2020 [19]	32	Antioquia	Recurrent proximal quadriparesis (UL 2/5, LL 1/5)	5 days	NR	1.6	NR	2.62	0.002	Normal	Methimazole + propranolol + cholestyramine, switched to PTU (no dose); I-131	No
Hoyos-Rizo et al., 2021 [20]	40	Santander	Paraparesis progressing to UL, with dysarthria and respiratory compromise	24 h (preceded by 1 month of myalgia)	LL hyporeflexia	1.90	NR	Elevated (no value)	Suppressed (no value)	NR	Methimazole + propranolol + prednisolone + potassium (no dose); I-131	Yes
Orjuela et al., 2022 [21]	33	Valle del Cauca	Ascending paraparesis (LL 1/5); UL preserved	7 days	LL hyporeflexia (UL normal)	1.7	2.06 (2.5 mg/dL)	3.3	<0.015	NR	Methimazole 20 mg every 12 h + propranolol 40 mg every 12 h (at discharge); potassium 10 mEq/h	Yes
Bustos Sánchez et al., 2024 (C1) [22]	31	Boyacá	Quadriparesis 4/5, LL predominance	4 days	Normal reflexes	3.1	NR	3.80	Undetectable	NR	Methimazole 10 mg every 12 h + potassium gluconate 10 mL every 8 h	No
	25	Casanare	Reported weakness with LL predominance; strength 5/5 on examination	4 days	Normal reflexes (DTR ++/++++)	1.9	NR	5.67	0.06	NR	Methimazole 10 mg every 12 h + propranolol 40 mg every 8 h + potassium gluconate	No
Holguín-Cardona et al., 2024 [23]	44	Bogotá	Asymmetric quadriparesis (left UL 2/5)	10 days (paresthesia and myalgia)	Normal reflexes (DTR ++/++++)	1.99	NR	5.14	<0.005	Sinus tachycardia, U waves	Methimazole 10→15 mg every 24 h + propranolol 40 mg every 12 h; potassium 8 mEq/h + MgSO_4_ 2 g every 12 h	No
Nati-Castillo et al., 2025 (index case)	38	Valle del Cauca	Nocturnal proximal quadriparesis (UL 4/5, LL 3/5)	18 h	Normal reflexes (DTR 2+)	2.3	1.6	>7.7	<0.005	QTc 649 ms, flattened T waves, prominent U waves	IV K^+^ (dose/rate unavailable); oral KCl 10%, 5 mL q8h × 7 days; methimazole 10 mg q12h; propranolol 40 mg q8h; IV methylprednisolone 1 g/day × 5 days, followed by prednisone 50 mg/day with taper	Yes

LL: lower limbs; UL: upper limbs; NR: not reported in the original publication; NV: not verifiable, as the full text could not be retrieved; PTU: propylthiouracil; ICU: intensive care unit; DTR: deep tendon reflexes. Reflex grades are presented as reported in the original publications: 0/++++ indicates absent reflexes, whereas ++/++++ indicates normal reflexes (equivalent to 2+). In this notation, the denominator ‘++++’ represents the maximum grade of the scale, rather than the patient’s observed reflex response. All 15 cases were male. Each cell reproduces only what the primary source states: where the original report did not specify a dose, a laboratory value or an examination finding, NR is recorded instead of an estimate. Ladino-Malagón et al. report total rather than free thyroxine, so those three values are not comparable with the rest of the column. (*) Data from Cogollo González et al. come from the published abstract; the full text remains behind an editorial subscription and could not be independently verified. Source: prepared by the authors. Time from onset of weakness to presentation is the interval between the onset of muscle weakness and the emergency department consultation, transcribed as stated in each primary report; no interval was inferred where the report did not state one.

Precipitating factors classically associated with TPP were documented in several of the Colombian reports: a carbohydrate-rich meal on the eve of every episode in one case and strenuous exercise twelve hours before onset in another [14], strenuous physical activity or an exhausting working day in the hours preceding the attack [16,17,21], and sustained alcohol intake [20]. Glucocorticoids deserve separate mention, because they increase skeletal-muscle Na^+^/K^+^-ATPase density and stimulate insulin secretion, and TPP has been reported after a single dose of prednisone [24]. In a Peruvian series of 22 patients, a carbohydrate-rich meal preceded the attack in 54% of cases and exercise in 27% [25]. Systematic documentation of exposure to these triggers is therefore warranted in future reports.

All reported Colombian patients recovered muscle strength following acute management. However, recurrence and long-term outcomes could not be reliably assessed because follow-up duration and completeness were inconsistently reported across cases. Therefore, the absence of documented recurrence should not be interpreted as evidence of uniformly recurrence-free outcomes. Clinically, TPP may occur in the absence of prominent manifestations of thyrotoxicosis, reinforcing the importance of considering thyroid dysfunction in patients presenting with otherwise unexplained acute proximal weakness and hypokalemia. Acute potassium replacement addresses the immediate electrolyte disturbance, whereas sustained control of the underlying thyrotoxicosis remains essential to reduce the risk of recurrent attacks.

Although the structured review was intentionally restricted to cases diagnosed in Colombia, three previously published Latin American cohorts or case series provide relevant regional context. A Peruvian series of 22 patients reported a mean age at diagnosis of 35.8 ± 9.6 years; the paralytic episode revealed previously unrecognized hyperthyroidism in 82% of patients, and Graves disease was identified as the underlying cause in 95% [25]. A Mexican series of 11 patients also showed male predominance, with hyperthyroidism known before the paralytic episode in only two patients [26]. In a Brazilian series of four patients, polymorphic KCNJ18 variants were detected in all participants, whereas no pathogenic variants were identified in CACNA1S or SCN4A [27]. These reports suggest that the demographic and clinical characteristics observed in the Colombian cases are broadly consistent with those described elsewhere in Latin America. Nevertheless, differences in study design, case ascertainment, sample size, and reporting completeness preclude direct comparisons between countries. This regional comparison was narrative rather than systematic and should not be interpreted as an exhaustive review of all reported Latin American cases.

The index patient was described in the medical record as Mestizo, a broad sociocultural category commonly used in Colombia for individuals of mixed ancestry. Similarly, all participants in the Peruvian series were classified as Mestizo by the original investigators [25]. However, this category does not represent a homogeneous genetic ancestry group and should not be interpreted as an independent marker of genetic susceptibility to TPP. Dietary potassium intake is also unlikely to explain the acute hypokalemia characteristic of TPP, which primarily results from a transcellular potassium shift rather than depletion of total-body potassium. In the index patient, no gastrointestinal potassium losses were reported, although quantitative dietary potassium intake was not assessed.

This review has several limitations inherent to the analysis of previously published case reports and small case series. Clinical and laboratory reporting was heterogeneous: deep tendon reflexes were not documented in two cases, ECG findings were available in only seven, serum magnesium was measured in four, and methimazole doses were reported in six. In addition, the full text of one publication was unavailable for independent verification, and data from that report were therefore derived from the published abstract and explicitly identified in Table 2. The small number of cases, potential publication bias, and absence of a national surveillance or registry system prevent estimation of incidence, prevalence, regional risk, or treatment effectiveness. Nevertheless, these reporting gaps highlight the need for more standardized documentation of TPP cases in Colombia. Future reports should incorporate established case-reporting frameworks such as the CARE guidelines and systematically document biochemical findings, electrocardiographic abnormalities, treatment, potassium replacement, recurrence, and longitudinal follow-up. Moreover, the comparison with the Peruvian, Mexican, and Brazilian series was included solely to provide regional context and was not based on a systematic search of the Latin American literature; consequently, additional regional reports may not have been captured.

## 5. Conclusions

Thyrotoxic periodic paralysis should be considered in patients presenting with acute proximal weakness and hypokalemia, particularly in young men, even when deep tendon reflexes are preserved or overt manifestations of thyrotoxicosis are absent. The published Colombian cases show a consistent clinical phenotype characterized by male predominance, marked hypokalemia, suppressed TSH, and recovery of muscle strength following acute management. However, substantial heterogeneity in the reporting of electrocardiographic findings, serum magnesium, treatment regimens, and follow-up limits more detailed characterization of complications, recurrence, and management patterns. Standardized reporting using frameworks such as the CARE guidelines, together with complete documentation of biochemical, electrocardiographic, therapeutic, and follow-up data, would strengthen future evidence on TPP in Colombia and other underrepresented populations.

## Figures and Tables

**Table 1:** Laboratory and diagnostic work-up.

Parameter	Result (Unit)	Reference Range
Complete blood count		
White blood cells	6850 (cells/mm^3^)	4000–11,000
Neutrophils	5069 (cells/mm^3^)	1500–8000
Lymphocytes	1342 (cells/mm^3^)	1000–4000
Eosinophils	150 (cells/mm^3^)	50–500
Hemoglobin	16 (g/dL)	13–17 (M)
Hematocrit	46 (%)	40–52 (M)
MCV	90 (fL)	80–100
Platelets	261,000 (mm^3^)	150,000–450,000
Acute-phase reactants		
ESR	10 (mm/h)	<20
CRP	0.06 (mg/dL)	<0.5
Infectious screening		
HIV	Non-reactive (-)	Non-reactive
VDRL	Non-reactive	Non-reactive
Hepatitis B surface antigen	Negative (-)	Non-reactive
Hepatitis C antibodies	Negative (-)	Non-reactive
Renal function		
Creatinine	0.74 (mg/dL)	0.7–1.3 (M)
BUN	11.6 (mg/dL)	7–20
eGFR (CKD-EPI)	123 (mL/min/1.73 m^2^)	>90
Urinalysis		
Specific gravity	1.015 (-)	1.005–1.030
pH	6 (-)	4.5–8
White blood cells	2 (per field)	<5
24-h urine		
Sodium	68 (mEq/day)	40–220
Potassium	14 (mEq/day) *	25–125
Creatinine	1200 (mg/24 h)	500–2000
Electrolytes		
Sodium	141 (mEq/L)	135–145
Potassium	2.3 (mEq/L) *	3.5–5.1
Chloride	107 (mEq/L)	98–107
Calcium	9.9 (mg/dL)	8.5–10.5
Magnesium	1.6 (mEq/L)	1.5–2.5
Phosphate	2.9 (mg/dL)	2.5–4.5
Arterial blood gases		
pH	7.35 (-)	7.35–7.45
pO_2_	85 (mmHg)	75–100
pCO_2_	38 (mmHg)	35–45
HCO_3_	24 (mmol/L)	22–26
Thyroid function		
Free T4	>7.7 (ng/dL) *	0.89–1.76
TSH	<0.005 (mIU/L) *	0.465–4.68
Metabolic		
Glucose	120 (mg/dL)	<100 (fasting)
HbA1c	<5.6 (%)	<5.7
CPK	42 (U/L)	30–200 (M)
Vitamins		
Vitamin B12	351 (pg/mL)	200–900
Folate	4.4 (ng/mL)	>3.0
ECG	Prolonged QTc (649 ms) *	QTc < 450 ms (M)

(*) Result outside the reference range. (M): reference range for men. Source: prepared by the authors.

## Data Availability

The original contributions presented in this study are included in the article. Further inquiries can be directed to the corresponding author.
